# Impact of the HIV-1 *env* Genetic Context outside HR1–HR2 on Resistance to the Fusion Inhibitor Enfuvirtide and Viral Infectivity in Clinical Isolates

**DOI:** 10.1371/journal.pone.0021535

**Published:** 2011-07-08

**Authors:** Franky Baatz, Monique Nijhuis, Morgane Lemaire, Martiene Riedijk, Annemarie M. J. Wensing, Jean-Yves Servais, Petra M. van Ham, Andy I. M. Hoepelman, Peter P. Koopmans, Herman G. Sprenger, Carole Devaux, Jean-Claude Schmit, Danielle Perez Bercoff

**Affiliations:** 1 Laboratory of Retrovirology, CRP-Santé, Luxembourg, Luxembourg; 2 Department of Virology, Medical Microbiology, UMC Utrecht, Utrecht, The Netherlands; 3 Department of Internal Medicine and Infectious Diseases, UMC Utrecht, Utrecht, The Netherlands; 4 Division Infectious Diseases, Department of General Internal Medicine, Radboud University Medical Center, Nijmegen, The Netherlands; 5 Division of Infectious Diseases, Department of Internal Medicine, University Medical Center Groningen, Groningen, The Netherlands; University of Stellenbosch, South Africa

## Abstract

Resistance mutations to the HIV-1 fusion inhibitor enfuvirtide emerge mainly within the drug's target region, HR1, and compensatory mutations have been described within HR2. The surrounding envelope (*env*) genetic context might also contribute to resistance, although to what extent and through which determinants remains elusive. To quantify the direct role of the *env* context in resistance to enfuvirtide and in viral infectivity, we compared enfuvirtide susceptibility and infectivity of recombinant viral pairs harboring the HR1–HR2 region or the full Env ectodomain of longitudinal *env* clones from 5 heavily treated patients failing enfuvirtide therapy. Prior to enfuvirtide treatment onset, no *env* carried known resistance mutations and full Env viruses were on average less susceptible than HR1–HR2 recombinants. All escape clones carried at least one of G36D, V38A, N42D and/or N43D/S in HR1, and accordingly, resistance increased 11- to 2800-fold relative to baseline. Resistance of full Env recombinant viruses was similar to resistance of their HR1–HR2 counterpart, indicating that HR1 and HR2 are the main contributors to resistance. Strictly X4 viruses were more resistant than strictly R5 viruses, while dual-tropic Envs featured similar resistance levels irrespective of the coreceptor expressed by the cell line used. Full Env recombinants from all patients gained infectivity under prolonged drug pressure; for HR1–HR2 viruses, infectivity remained steady for 3/5 patients, while for 2/5 patients, gains in infectivity paralleled those of the corresponding full Env recombinants, indicating that the *env* genetic context accounts mainly for infectivity adjustments. Phylogenetic analyses revealed that quasispecies selection is a step-wise process where selection of enfuvirtide resistance is a dominant factor early during therapy, while increased infectivity is the prominent driver under prolonged therapy.

## Introduction

The human immunodeficiency virus type 1 (HIV-1) envelope glycoprotein (Env) mediates viral entry into the host cell by fusion of the viral envelope with the host cell membrane (reviewed in [1], [2], [3]). The Env complex is composed of two non-covalently linked subunits, the surface glycoprotein (gp120) and the transmembrane glycoprotein (gp41), displayed as homotrimers at the surface of the virion and of infected cells. Viral entry is a multistep phenomenon: Binding of gp120 to the CD4 receptor expressed on the surface of target cells induces a conformational change that exposes the third hypervariable loop (V3) of gp120, which in turn binds one of the two chemokine receptors CCR5 (R5 viruses) or CXCR4 (X4 viruses). Consequently, the viral V3 sequence defines cell tropism to a large extent, although regions outside the V3-loop have been described to modulate coreceptor usage [4], [5], [6]. Coreceptor binding triggers further conformational changes in the ectodomain of gp41 that lead to the insertion of the N-terminal glycine-rich fusion peptide into the host cell membrane. Folding of heptad repeat 2 (HR2) region onto heptad repeat 1 (HR1) region forms a highly stable six helix bundle structure and brings the viral and host cell membranes into close contact, ultimately leading to the fusion of both membranes [7], [8], [9], [10].

It has been shown that synthetic peptides that bind to one of the HR motifs interfere with the formation of a stable six helix bundle and inhibit viral entry [7], [11], [12], [13]. Enfuvirtide (ENF, T-20) [11], [14] is a subcutaneously injected 36 amino acids (AA) peptide mimicking part of the HR2 sequence (AA 127 to 162). Enfuvirtide binds HR1 and hinders the fusion process by preventing the HR1–HR2 interaction. Enfuvirtide is active against multi-drug resistant viral strains and is currently recommended as salvage therapy for highly drug experienced patients.

Enfuvirtide resistant HIV-1 variants however rapidly emerge under enfuvirtide selective pressure [15], [16] and resistance mutaftions have been described both *in vitro* and *in vivo* [17], [18], [19], [20], [21], [22], [23], [24], [25], [26], [27]. Most resistance mutations are located within HR1, between AA 36 and 45 (HXB_2_ numbering) [17], [18], [25]. Some resistance mutations, reduce viral infectivity in the absence of the drug, probably as a consequence of impaired interactions between HR1 and HR2 and delayed fusion kinetics [19], [28], [29], [30]. Compensatory mutations that restore viral infectivity may arise within HR2 [19], [26], [29], [31]. Some of these mutations, such as the S138A substitution, have been suggested to confer some resistance *per se* [19], [29], but others do not or only modestly impact the level of resistance.

Enfuvirtide operates by a unique mechanism as it does not target the static Env, but a structural intermediate of the entry process, called the fusogenic intermediate, induced by binding of gp120 to the CD4 receptor. Hence, factors that influence the short kinetic window during which HR1 is accessible to the peptide also influence viral susceptibility. Env determinants outside of HR1 and HR2, including tropism, coreceptor affinity [30], [32], [33], the CD4 binding region of gp120 [20] and the bridging sheet region [30], have been shown to modulate the level of susceptibility/resistance to enfuvirtide. Although the impact of coreceptor usage on the level of susceptibility has been addressed by many authors, results remain controversial, likely a consequence of different experimental and analytical approaches [27], [32].

Furthermore, it has been suggested that other determinants outside of HR1 may be involved in resistance and/or that the *env* genetic context drives the selection of Envs in which resistance mutations emerge [34], [35], [36]. The present study addresses the relative contributions of Env determinants outside of the HR1 and HR2 regions to resistance to enfuvirtide and to viral infectivity by comparing NL4-3-derived recombinant virus pairs harboring either the sole HR1–HR2 region or the entire Env (gp120+gp41) ectodomain from longitudinal HIV-1 Envs cloned from plasma from 5 heavily treated patients receiving enfuvirtide as part of a salvage regimen. Our results indicate that (i) the HR1–HR2 region is the major contributor to resistance, while the *env* genetic background modulates baseline susceptibility and to a lesser extent resistance after virological failure, (ii) coreceptor usage, and particularly strict X4 tropism, was associated with higher resistance (iii) the *env* genetic context contributes to restoring viral infectivity, and (iv) both the level of resistance and viral infectivity orchestrate the selection of variants under prolonged enfuvirtide pressure.

## Results

### Generation of HR1–HR2 and full Env recombinant viral particles

Eighty full envelopes were cloned. All the tested HR1–HR2 recombinant viruses were infectious, but only 65% (52/80) of the full Env recombinant viruses were infectious, in line with previous reports [35]. A higher proportion (77.3%, 17/22) of baseline clones were infectious compared to enfuvirtide-escape clones (60.3%, 35/58) and very few late clones were infectious (Table S1). Phenotypic analyses were restricted to clones that allowed to generate both HR1–HR2 and full Env infectious recombinant viruses (n = 52).

### Baseline genotypic and phenotypic susceptibility to enfuvirtide

No known enfuvirtide resistance mutation was detected in pre-treatment clones. Viruses from patients A, C, D and E carried the subtype B HR1 consensus sequence GIVQQQNNLL between AA 36 and 45. All the pre-treatment viral *env* clones from patient B carried the N42S polymorphism (Table S1).

**Figure 1:**
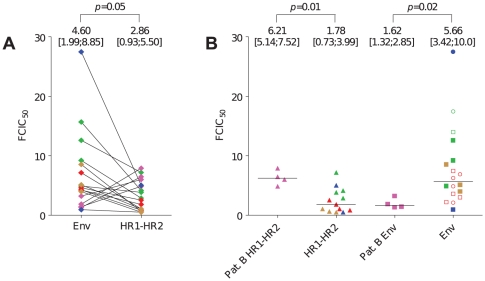
Baseline fold changes in enfuvirtide IC_50_ (FCIC_50_) of the full Env and HR1–HR2 recombinant viruses from patients A (green), patient B (pink), patient C (red), patient D (blue) and patient E (brown) determined on U87.CD4 cells expressing either CCR5 or CXCR4. Medians with interquartile ranges are reported at the top of the graphs. **Panel A**: Pairwise comparison of full Env FCIC_50_ and HR1–HR2 FCIC_50_ for each patient. **Panel B**: FCIC_50_ of the HR1–HR2 (column 1) and full Env recombinant viruses (column 3) from patient B compared to the HR1–HR2 (column 2) and full Env recombinant viruses (column 4) from all other patients. HR1–HR2 viruses are represented as closed triangles (▴). For full Env recombinant viruses, closed circles (•) represent strictly X4 viruses, open circles (○) represent dual-tropic clones tested on CXCR4-expressing cells, closed squares (▪) represent strictly R5 viruses and open squares (□) represent dual-tropic clones tested on CCR5-expressing cells.

**Figure 2:**
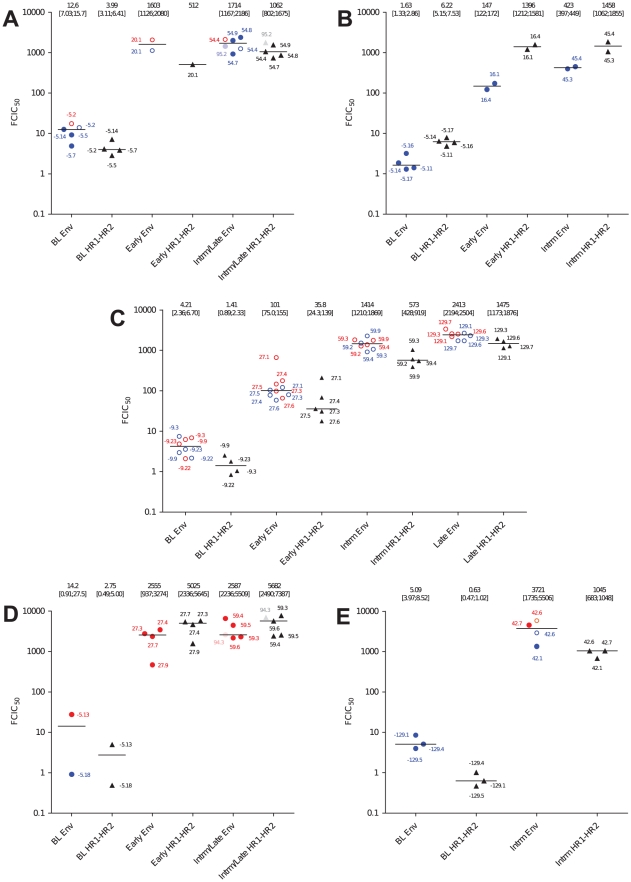
Phenotypic enfuvirtide resistance of full Env and HR1–HR2 recombinant viruses. U87.CD4.CCR5 and U87.CD4.CXCR4 were infected with HR1–HR2 and full Env recombinant viruses in the presence of decreasing concentrations of enfuvirtide, ranging from 15×10^3^ ng/ml to 0.04 ng/ml). Resistance is expressed as fold changes in IC_50_ (FCIC_50_) normalized to the HXB_2_ reference. For full Env recombinant viruses, resistance was determined on U87.CD4 cells expressing either CCR5 (red) or CXCR4 (blue). Closed circles (•) represent strictly R5 or X4 recombinant viruses and open circles (○) represent dual-tropic recombinant viruses. FCIC_50_ of HR1–HR2 recombinant viruses are represented as closed triangles (▴) and were tested on U87.CD.CXCR4 cells. Medians (horizontal bars) of at least two independent experiments are shown. Medians with interquartile ranges are reported at the top of the graphs.

Full Env recombinant viruses displayed greater variability in enfuvirtide susceptibility (FCIC_50_ range: 0.91–27.47) than HR1–HR2 recombinant viruses (FCIC_50_ range: 0.47–7.16) (Fig. 1.A). Overall median FCIC_50_ of the full Env recombinant viruses was higher than that of the HR1–HR2 recombinant viruses (Fig. 1.A) (*p* = 0.05), indicating that the *env* genetic context lowered susceptibility to enfuvirtide. At a single clone level, all the full Env pre-treatment recombinant viruses from patients A, C, D and E were less susceptible than the corresponding HR1–HR2 recombinant viruses (Fig. 1.A and Fig. 2.A, C, D and E, Table S1).

In contrast, patient B pre-treatment HR1–HR2 recombinant viruses displayed higher FCIC_50_ than their full Env counterparts (Fig. 1.A). When compared to the 4 other patients' *envs*, full Env recombinant viruses from patient B were more susceptible to enfuvirtide than those from the 4 other patients (*p* = 0.02), while HR1–HR2 recombinant viruses were less susceptible than those of the 4 other patients (*p* = 0.01) (Fig. 1.B).

### Genotypic and phenotypic resistance during enfuvirtide treatment

All the clones retrieved from patients after enfuvirtide treatment failure harbored at least one known resistance mutation within HR1, at positions 36 and/or 38 (patients A and C) and at positions 42 and/or 43 (patients B, D and E) (Table S1). Of note, none of the clones carried resistance mutations concomitantly at positions 36 and 38, 36 and 43 or 38 and 43, in contrast to reports by others [34], [35]. FCIC_50_ of escape recombinant viruses increased 11- to 2800-fold relative to baseline. Overall, median FCIC_50_ of full Env recombinant viruses did not differ significantly from the FCIC_50_ of HR1–HR2 recombinant viruses (full Env median FCIC_50_ = 1469 [177;2401], HR1–HR2 median FCIC_50_ = 1062 [481;1877], *p*>0.05). Likewise, at an individual patient level, no significant difference was detected in the level of resistance between full Env and HR1–HR2 recombinant viruses (patients A, B, D and E).

For 3 patients, resistance was associated with mutations at one position at all time points (AA 36 for patient A, AA 43 for patients B and E) and featured an over 100-fold increase in FCIC_50_ relative to the corresponding baseline clones, regardless of coreceptor usage (Fig. 2.A, B and E, Table S1). For patient B, FCIC_50_s of the HR1–HR2 recombinant viruses were higher than those of the corresponding full Env recombinant viruses at weeks 16 and 45, as for baseline clones. Furthermore, the level of resistance of HR1–HR2 recombinant viruses remained steady between weeks 16 and 45, whereas full Env recombinant viruses reached 3-fold higher resistance levels at week 45 than at week 16 (Fig. 2.B, Table S1), highlighting that the *env* genetic context did not easily tolerate the constraints that allowed escaping enfuvirtide treatment. Because of the limited availability of early and late clones for patients A and E, variations in the level of resistance over time could not be assessed.

For patients C and D, resistance was associated with multiple mutations evolving over time. For patient C, 4/5 early escape viruses carried the G36D substitution and one clone carried the V38A mutation. The V38A variant featured 4-fold (full Env) and 6-fold (HR1–HR2) higher resistance than the early G36D clones (Fig. 2.C, Table S1). At later time points, only the V38A mutation was detected associated with the L44M resistance mutation. Resistance of intermediate and late clones increased by 15-fold at week 59, and by 40-fold (HR1–HR2 recombinants) and 24-fold (full Env recombinants) at week 129 (*p* = 0.03). For patient D, 3/5 early *env* clones carried the N43D resistance mutation and one infectious clone carried the N42D+N43S mutations (Fig. 2.D, Table S1). The N43D mutants were 2 to 6-fold more resistant than the N42D+N43S double mutant for both HR1–HR2 and full Env recombinants (Fig. 2.D, Table S1). All intermediate and late Env clones (weeks 59 and 94) carried the N43D mutation with the N126K substitution [37], although in contrast to patient C, phenotypic resistance levels did not increase further under prolonged treatment (Fig. 2.D and Table S1). These observations suggest that the higher level of resistance conferred by the V38A or the N43D mutations drove the selection of Envs between early and intermediate/late time points.

Altogether, HR1–HR2 contains the main determinants of resistance, irrespective of the resistance mutations that were selected, while other *env*-encoded determinants outside of HR1–HR2 modulate the level of resistance to a marginal extent, probably by driving the selection process of viral Envs with increased resistance under drug pressure.

### Impact of tropism on the level of resistance

Because tropism has been described to modulate susceptibility to enfuvirtide [27], [32], we compared resistance of strictly X4, strictly R5 and dual tropic clones. X4 Env recombinant viruses tended to be more resistant than R5 recombinant viruses at baseline and after virological failure (2.7-fold) (X4 median FCIC_50_ = 2499 [2273;4464], R5 median FCIC_50_ = 924 [284;1714]) (*p* = 0.001). For dual-tropic viruses, resistance was found to be similar, irrespective of the coreceptor expressed by the cell line used (data not shown).

### Relative viral infectivity during enfuvirtide treatment

We then investigated whether the *env* genetic context surrounding HR1–HR2 played a role in infectivity adjustments. Overall, HR1–HR2 recombinant viruses featured higher median relative infectivities (RI) than their full Env counterparts prior to and after enfuvirtide treatment escape: pre-treatment median HR1–HR2 RI = 0.89 [0.58;1.22] vs median Env RI = 0.16 [0.04;0.43], (*p* = 0.006) and after treatment failure median HR1–HR2 RI = 0.67 [0.44;1.21] vs median Env RI = 0.28 [0.13;0.75], *p* = 0.01. Because in this *in vitro* system differences in infectivity between paired recombinant viruses probably reflect the higher infectivity of HXB_2_-derived gp120 of HR1–HR2 recombinants over patient-derived primary gp120 of full *env* recombinants, we compared the changes in RI of HR1–HR2 and of full Env recombinant viruses during the time of follow-up rather than HR1–HR2/full Env recombinant virus pairs at a given time point.

**Figure 3:**
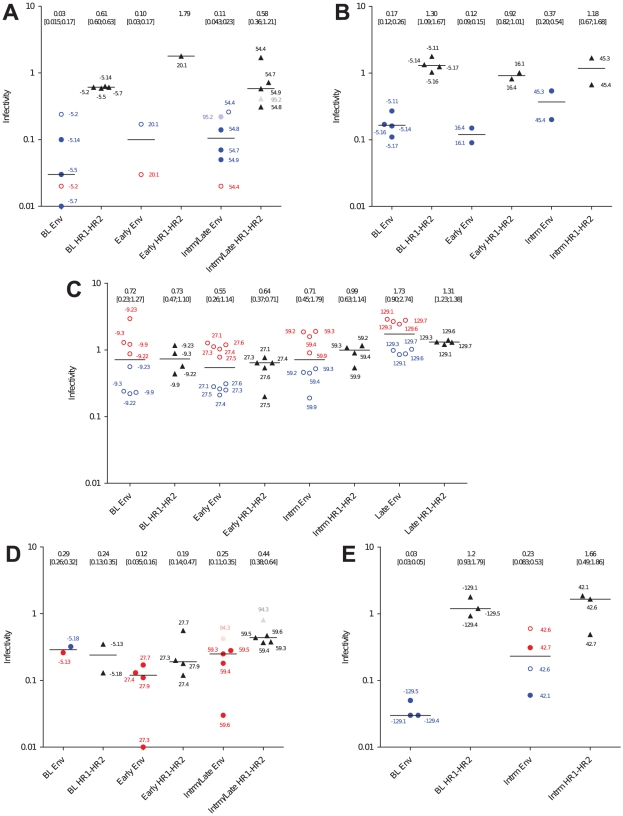
Relative infectivity (RI) of full Env and HR1–HR2 recombinant viruses. U87.CD4.CCR5 and U87.CD4.CXCR4 were infected with serial two-fold dilutions (ranging from 400 pg p24/well to 50 pg p24/well) of HR1–HR2 and full Env recombinant viruses. Luciferase activity was monitored as a function of p24 input to assess infectivity and was normalized to the HXB_2_ reference to estimate RI. For full Env recombinant viruses, RI was determined on U87.CD4 cells expressing either CCR5 (red) or CXCR4 (blue). Closed circles (•) represent strictly R5 or X4 recombinant viruses and open circles (○) represent dual-tropic recombinant viruses. RI of HR1–HR2 recombinant viruses are represented as closed triangles (▴) and were tested on U87.CD.CXCR4 cells. Medians (horizontal bars) of at least two independent experiments are shown. Medians with interquartile ranges are reported at the top of the graphs.

RIs of full Env recombinant viruses spanned a wider range than RIs of HR1–HR2 recombinant viruses, consistent with the high genotypic conservation of the HR1–HR2 region. Early after enfuvirtide failure, RI of the escape variants was similar to or lower than at baseline for both HR1–HR2 and full Env recombinant viruses (Fig. 3). At later time points (intermediate and late), RI of full Env recombinant viruses increased for all patients relative to early escape clones (Fig. 3). The RI of HR1–HR2 recombinant viruses, by contrast, remained steady for patients A, B and E, strongly indicating that determinants other than HR1–HR2 account for infectivity adjustments, either through the emergence of compensatory mutations or through the selection of Envs that best tolerate resistance mutations. For patients C and D, in contrast, HR1–HR2 recombinant viruses showed a trend to increase, likely mapping determinants that account for gains in infectivity to HR1–HR2.

Among the polymorphisms previously described to modulate viral infectivity, compensatory mutation S138A [19] was detected in 5/9 escape Envs from patient B, in 9/10 intermediate and late escape Envs from patient D (but in none of the early escape Envs) and in all viral escape clones from patient E (Table S1). In all patients, however, the RIs of variants carrying the S138A mutation were comparable to those of variants carrying wild-type S138 (Fig. 3 and Table S1), suggestive that the relative contribution of the S138A substitution to infectivity rescue might also involve other determinants. Other mutations included substitutions N125D [29], [38] (patients A, B and C), N125S and N126K [37], [39], [40] (patient D) and E137K [41], [42] (patient E) (Table S1). The N125D/S polymorphisms within HR2 were detected in both baseline and escape clones, arguing against them playing a pivotal role in restoring infectivity. For patient E, the impact of E137K is difficult to evaluate as only intermediate clones were recovered and as gains in viral infectivity seem to map to *env* determinants outside of HR1–HR2 (Fig. 3.E). In contrast, the N126K compensatory mutation in patient D viruses was detected exclusively in on-treatment viral sequences, pointing to it as a presumed contributor to the parallel infectivity gains of HR1–HR2 and full Env recombinants. Taken together, our data suggest that both compensatory mutations within HR1–HR2 (N126K for patient D, yet unidentified determinants for patient C) and the *env* genetic context (patients A, B and E) contribute to increased viral infectivity.

### Phylogenetic analysis of full *env* sequences

Phylogenetic analyses were performed to dissect the relative contributions of resistance and of infectivity to selective evolution under prolonged enfuvirtide pressure.

**Figure 4:**
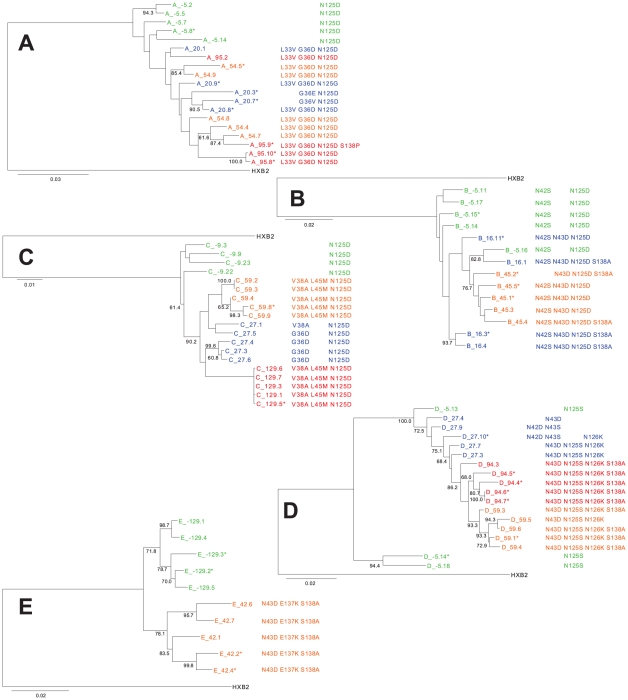
Phylogenetic analysis of the full *env* sequences. Complete *env* coding sequences were aligned with HXB_2_ as the outgroup using the MUSCLE software and phylogenetic trees were constructed using the PhyML software. Main resistance mutations and compensatory mutations are indicated for each clone. Non-infectious clones are marked with a *. Bootstrap values >60% are indicated.

For patients A and B, all the clones (baseline and post-treatment failure) were intermingled, suggesting that these viral populations explored different evolutionary routes. Early resistance mutations (G36D and N42S+N43D) conferred high level resistance (>100-fold) and persisted throughout treatment (Fig. 4.A and B). For patient B, week 45 clones grouped on one branch and were related to pre-treatment clone −5.16 and early clone 16.1. The gain in infectivity of intermediate clones relative to the early clones was higher than the gain in resistance, suggesting that the evolutionary paths embraced must have favored infectivity adjustments.

For patients C and D, viral clones from each time point clustered together. Both patients hosted strains with resistance mutations at 2 positions at early time points (Table S1), and determinants within HR1–HR2 were sufficient to confer enfuvirtide resistance and to restore viral infectivity. The V38A (patient C) and N43D (patient D) mutations conferred higher resistance than the G36D (patient C) or N42D+N43S (patient D) mutations respectively, and at intermediate and late time points, the early G36D and N42D+N43S variants were outcompeted by V38A or the N43D variants (Fig. 2 and Fig. 3), indicating that early after treatment escape, the level of resistance is the main driver of viral selection.

It is noteworthy that for patients A and D, only one late clone was infectious. For patient C, all late (week 129) clones were extremely tightly related and emerged from the branch grouping early G36D variants (clones 27.3, 27.4, 27.6) rather than from the early V38A variant or from the intermediate V38A+L45M variants (Fig. 4.C). For these 3 patients, late infectious clones stemmed from early rather than from intermediate escape clones, suggesting that the other evolutionary viable paths explored by the virus at the intermediate time point, led to a dead-end under pursued enfuvirtide pressure (Fig. 4.A, C and D). Late infectious clones had similar resistance levels than early/intermediate clones and high RIs (Fig. 2 and Fig. 3), indicating that infectivity levels molded selection at late time points.

These findings highlight restricted evolution and limited evolutionary possibilities under prolonged enfuvirtide pressure, and illustrate that while resistance may be achieved through different paths, optimal Env properties enhancing RI are highly constrained.

## Discussion

In this study, direct comparison of HR1–HR2 and full Env recombinant viral particles containing longitudinal Envs from 5 patients receiving prolonged enfuvirtide-based therapy, formally points to HR1 and HR2 as the principal contributors to high level resistance. The surrounding *env* genetic context played a modulatory role on basal susceptibility and to a lesser extent on the level of resistance, in line with a previous report [16]. Previous studies have suggested that the *env* genetic context contributes to resistance to enfuvirtide both directly and by driving the selection of resistance mutations [23], [34], [35]. In these reports, escape variants that appeared under enfuvirtide pressure evolved from minority variants present prior to treatment. Because the selected resistance mutations conferred higher resistance levels within the genetic context within which they arose than in pre-therapeutic *env* clones from the same patient, the authors conclude that the *env* genetic context drives the resistance pathway embraced by viruses under drug pressure and that the level of resistance is the primary determinant of selection [34], [35]. These reports contrast with the mainly modulatory role of the *env* genetic context we recorded. In our study, the weight of the full Env ectodomain was compared to isogenic HR1–HR2, while in the study by Goubard *et al.* [35] one single mutation (V38A) was inserted by site-directed mutagenesis. It is therefore not possible to exclude that in their study, the HR1–HR2 region rather than the full Env accounted for the *env*-encoded contribution to enfuvirtide resistance.

In the case of patient B, the *env* genetic context lowered the level of resistance. Furthermore, the HR1–HR2 recombinant viruses from this patient were less susceptible to enfuvirtide than the HR1–HR2 recombinants from the other patients despite the N42S polymorphism, which has been associated with increased enfuvirtide susceptibility and a slightly improved virological outcome [25], [27], [43]. In this patient, other yet undetermined, *env*-encoded determinants likely contributed to the particularly high susceptibility to enfuvirtide, and/or the *env* genetic context remained essentially unfavorable to the development of enfuvirtide resistance. Noteworthingly, patient B hosted only strictly R5 viruses. It is therefore possible that tropism was one of the determinants that contributed to render full Env recombinants less susceptibile to enfuvirtide than the isogenic HR1–HR2 recombinants.

We show that X4-coreceptor usage was the only factor associated with significantly lower resistance levels. Coreceptor usage has been previously reported to influence susceptibility/resistance to enfuvirtide, but contradictory results have been reported depending on the design of the study and on whether dual/mixed viruses are considered separately or included among the X4 strains [27], [32], [33], [36]. Here we constructed *env* recombinant clones and analyzed strictly R5, strictly X4 and dual-tropic viruses as 3 distinct groups. The lower susceptibility of X4 recombinant viruses at baseline and after virological failure may reflect other intrinsic properties of gp120 [30], [44], [45]. Formal quantification of the gain in resistance associated with X4 tropism would require switching tropism by site-directed mutagenesis. In our study, X4 recombinant viruses (both full Env X4 and HR1–HR2 viruses) also featured higher infectivity than R5 recombinants. Although the design of our experimental system is not suited to study the potential relationship between the level of resistance and infectivity, it is plausible that the lower susceptibility of X4 recombinant viruses is related to properties of gp120 that accelerate the fusion process, such as fusion kinetics, coreceptor affinity and binding sites [30], [44], [45]. Yet, the fact that dual tropic full Env recombinant viruses displayed similar FCIC_50_ in both the CXCR4- and CCR5-cell lines irrespective of infectivity levels in each cell type, argues against a direct relation between viral infectivity and enfuvirtide susceptibility. Alternatively, dual tropic viruses might differ from strictly R5 or strictly X4 viruses through intermediate fusion kinetics, in line with their intermediate median FCIC_50_ (data not shown).

Previous studies have reported both decreases and increases in viral infectivity under enfuvirtide treatment [28], [34]. We did not detect pronounced reductions in viral infectivity (for both HR1–HR2 and full Env recombinant viruses), as reported by others [23]. However, working with clones implicitly restrains phenotypic analyses to infectious clones only, and we cannot exclude that losses in viral infectivity might be underestimated. Indeed, 77.3% of the baseline clones but only 60.3% of the escape clones were infectious, especially very few late clones were infectious. We found that both determinants within the HR1–HR2 regions (including compensatory mutations such as the S138A, N125D or N126K) and other gp120 properties likely accounted for gains in viral infectivity.

All patients achieved high level resistance as of the earliest time points and different resistance pathways were attempted. Early escape clones (patients C and D) with lower resistance were outcompeted by more resistant clones carrying the V38A or N43D resistance mutations respectively, indicating that the level of resistance reached is indeed one major determinant of evolution, as previously suggested [15], [23], [35]. Nonetheless, we found that intermediate and late infectious clones from all patients progressively gained RI revealing that viral infectivity also strongly molds the selection of escape variants, and becomes the prevalent actor of evolutionary adjustments under prolonged drug pressure once high level resistance is established. These apparently discrepant results with previous reports that attribute the selection of viral strains under sustained enfuvirtide pressure to the sole level of resistance [23], [35] might be due to the duration of enfuvirtide pressure: the latest on-therapy samples in the studies by Menzo *et al.* and by Goubard *et al.* were retrieved after 20 and 37 weeks of treatment, corresponding to a time-point between early and intermediate in our study. At these time-points, the level of resistance is indeed probably the major determinant of quasispecies selection, and viral diversity is still preserved. However, at later time points it is the level of infectivity that drives the selection of particular Envs, a process that is highly constrained and strikes genetic diversity.

In conclusion, our findings indicate that selection of viral variants is molded gradually by the level of resistance achieved through mutations within HR1–HR2 at first, and by the viral determinants mining viral infectivity, including specific compensatory mutations and polymorphisms as well as other Env properties. The first step of the process, escaping enfuvirtide pressure and achieving resistance, can occur via different routes and involve a diversity of mutations, as illustrated by the relative dispersal of early escape clones from all patients throughout the phylogenetic trees. Infectivity adjustments occur later and are likely more strongly constrained by the *env* genetic context, translating into evolutionary “turn-backs” and scarse phylogenetic diversity.

## Materials and Methods

### Patients and patient samples

Patients belong to the Dutch ATHENA Cohort for treatment evaluation and the study received ethical approval in the Netherlands. HIV-infected patients are informed that their data/samples are collected as part of the ATHENA evaluation study and consent is arranged according to an opting out procedure. Clinicians sign a form to assign patients to the Cohort.

Five heavily treated male patients receiving enfuvirtide (90 mg twice daily) (Roche Pharmaceuticals) as part of a salvage regimen in addition to an optimized background treatment (OBT) and who experienced virological failure were selected. All patients were infected with subtype B viruses. Median viral load was 125372 RNA copies/ml (range: 4960–372000) and mean CD4^+^ cell counts were 147 cells/µl (range: 30–410) at baseline (Table S2). A median of 10 (range: 9–12) resistance mutations to reverse transcriptase inhibitors and a median of 10 (range: 6–12) resistance mutations to protease inhibitors were detected. Enfuvirtide treatment initiation induced a decrease from 0.8 log to 3.0 log HIV-RNA. All patients experienced virological failure after a median of 20 weeks on enfuvirtide+OBT. Patient B interrupted ENF therapy between weeks 22 and 29. Longitudinal frozen plasma samples prior to enfuvirtide treatment (baseline) and longitudinal samples during treatment failure were selected.

### Cell lines

HEK293T (ATCC) cells were maintained in Dulbecco's Modified Eagle Medium (DMEM) supplemented with 10% heat-inactivated foetal bovine serum (FBS), L-glutamine (2 mM), penicillin (50 U/mL) and streptomycine (50 µg/mL) (all from Gibco). U87.CD4.CCR5 and U87.CD4.CXCR4 cells (AIDS Research and Reference Reagent Program, Division of AIDS, NIAID, NIH [46]) were maintained in DMEM supplemented with 15% FBS, L-glutamine (2 mM), penicillin (100 U/mL), streptomycine (100 µg/mL), geneticine (300 ng/mL) (Gibco) and puromycine (1 ng/mL) (Sigma-Aldrich). Expression of CD4, CCR5 and CXCR4 was monitored by flow cytometry.

### Cloning and analysis of patient-derived full *env*s

HIV-1 RNA was isolated as previously described [47]. *env* was reverse transcribed and amplified using forward primer Env1a (5′-GGCTTAGGCATCTCCTATGGCAGGAAGAA-3′ HXB_2_ 5954-5982) and reverse primer Env1b (5′-TAGCCCTTCCAGTCCCCCCTTTTCTTTTA-3′ HXB_2_ 9096-9068) for patient C (baseline, early and intermediate) and forward primer FLenv1.1 (5′-TAGAGCCCTGGAAGCATCCAGGAAG-3′ HXB_2_ 5853-5877) and reverse primer FLenv1.2 (5′-TTGCTACTTGTGATTGCTCCATGT-3′ HXB_2_ 8936-8913) for all other samples The RT-PCR mix contained. 4.8 µl viral RNA, 0.2 µM of each primer, 0,4 mM of each dNTP (GE HealthCare), 3.3 mM MgSO_4_, 0.5 µl SuperScript III RT/Platinum *Taq* High Fidelity DNA polymerase and 8 units RNAseOUT (Invitrogen Life Sciences), and conditions were as follows: reverse transcription for 30 min at 55°C, denaturation for 2 min at 94°C, and 30 amplification cycles (94°C for 20 s, 55°C for 30 s, 68°C for 4 min) followed by a final extension at 68°C for 5 minutes. 5 µl of this PCR product were further amplified over 30 cycles (95°C for 15 s, 58°C for 30 s, 68°C for 4 min) using forward primer Env2a (5′-AGAAAGAGCAGAAGACAGTGGCAATGA-3′) HXB_2_ 6202-6228) and reverse primer Env2b (5′-TTTTGACCACTTGCCACCCAT-3′ HXB_2_ 8797-8817) for patient C (baseline, early and intermediate) and forward primer FLenv2.1 (5′-GATCAAGCTTTAGGCATCTCCTATGfGCAGGAAGAAG-3′ HXB_2_ nt 5957-5983) and reverse primer FLenv2.2 (5′-AGCTGGATCCGTCTCGAGATACTGCTCCCACCC-3′ HXB_2_ 8904-8882) for all other samples. The nested PCR mix contained 2.0 mM MgSO_4_, 0.3 µM of each primer, 1.6 units Expand High Fidelity enzyme mix (Roche Applied Science) and 0,2 mM of each dNTP in Expand High Fidelity buffer. PCR products were purified (QIAquick PCR purification kit, Qiagen), A-tailed with 5 units of *Taq* DNA polymerase (Roche Applied Biosystems) at 70°C for 20 minutes, and TA-cloned in the pGEM-T-easy vector (Promega) using T4 DNA ligase (Promega) overnight at 4°C. Five clones were randomly selected for each time point (Table S1). For data analysis purposes, clones were classified as “baseline” (pre-enfufvirtide), “early” (first time point after virological failure, weeks 16 to 27), “intermediate” (weeks 42 to 59) and “late” (after week 94). For patients A and D, only one late clone was infectious and was therefore grouped with the corresponding intermediate clones.

All clones were sequenced (Genbank accession numbers HQ386140 to HQ386219). Full clonal *env* sequences were aligned against HXB_2_ using the MUSCLE software (v3.8.31) [48].

### Amplification of patient-derived *env* and *HR1–HR2* sequences

*env* and *HR1–HR2* fragments were PCR amplified from single clones and from reference pHXB_2_-env (AIDS Research and Reference Reagent Program, NIH) [49] using forward primer FLenv2.11. (5′-GCTTAGGCATCTCCTATGGCAGGAAGAAG-3′ HXB_2_ 5955-5983) and reverse primer rec.env_RP (5′-AAGCCCTGTCTTATTCTTCTAGGTATGT-3′ HXB_2_ 8776-8749) for the *env* amplicon, and forward primer rec.HR1-2_FP (5′-GAGGGACAATTGGAGAAGTGAAT-3′ HXB_2_ 7649-7671) and reverse primer rec.HR1-2_RP (5′-GGTGAATATCCCTGCCTAACTC-3′ HXB_2_ 8365-8344) for the *HR1–HR2* amplicon. Clonal template DNA was amplified using 1.5 units Platinum *Taq* High Fidelity DNA polymerase (Invitrogen Life Sciences) in High Fidelity Platinum PCR buffer containing 1,5 mM MgSO_4_, 0,2 mM of each dNTP, 0,2 µM of each primer over 35 cycles (94°C for 30 s, 64°C for 30 s, 68°C for 3 min (*env*)/1 min (*HR1–HR2*)) followed by a final extension at 68°C for 10 minutes. DNA concentration and quality were assessed by gel electrophoresis and triplicate DNA amplifications were pooled.

### Phylogenetic analyses

Phylogenetic analyses of *env* sequences were performed using the Maximum Likelihood method [50], [51] and the gamma corrected HKY85 [52] model of molecular evolution as suggested by the TOPALi software package (v2.5), setting HXB_2_ as the outgroup. Bootstrap values were determined using 1000 replicates.

### Production of Env and HR1–HR2 recombinant viruses

The *Eco*RI-*Not*I fragment from the previously described pNL4-3.GIV.eGFP [53], was subcloned into pZero-2 (Invitrogen Life Sciences) to engineer two luciferase-tagged pNL4-3-derived vectors pNL4-3Δ*env* and pNL4-3Δ*HR1–HR2*. To construct pNL4-3Δ*env*, the *env* ectodomain was deleted by inverse PCR using the phosphorylated reverse primer del.env_RP (5′-*GCT*GTCTTCTGCTCTTTCTATTAG-3′, HXB_2_ 6220-6197) and forward primer del.env_FP (5′-*GCT*GTACTTTCTATAGTGAATAGAGTT-3′, HXB_2_ 8322-8348), creating an *Afe*I restriction site (shown in *italic*) for linearization. The parental DNA fragment was *Dpn*I digested and the PCR amplicon was self-ligated using T4 DNA ligase (New England Biolabs) and transformed into electrocompetent TOP10 *E.coli* cells (Invitrogen Life Sciences). The *Sal*I-*Bam*HI fragment containing the remaining endodomain of gp41 was then cloned into the *Sal*I-*Bam*HI digested plasmid pNL4-3.Luc (gift of T. Dragic, Albert Einstein College of Medicine) [54], generating pNL4-3Δ*env*.Luc. pNL4-3Δ*HR1–HR2*.Luc was engineered similarly, using the phosphorylated reverse primer del.HR1-2_RP (5′-ATT*GGCGCGCC*TGTACCGTCA-3′ (HXB_2_ 7854-7834) and forward primer del.HR1-2_FP (5′-GATAAATGGGCAAGTTTGTGG-3′ (HXB_2_ 8214-8234) creating an *Asc*I restriction site (shown in *italics*) (Fig. S1) for linearization. Correct deletion of the *env* and of the *HR1–HR2* regions was attested by sequencing.

Full Env and HR1–HR2 recombinant viral particles were generated by cotransfecting HEK293T cells with 8 µg of *AfeI*-linearized pNL4-3Δ*env*.Luc or *AscI*-linearized pNL4-3Δ*HR1–HR2*.Luc with 4 µg of *env* amplicon or 1 µg of *HR1–HR2* amplicon respectively, packaged in 20 µl of HEKFectine (Bio-Rad). Cell culture medium was harvested 48 hours later and clarified at 4°C to eliminate cell debris, and p24 antigen concentration was quantified by ELISA (Perkin-Elmer).

Experimental biases due to the recombination event with two different amplicons were excluded by verifying that HR1–HR2 and full Env recombinant viruses produced from HXB_2_ and from an otherwise identical HXB_2_-derived double mutant carrying the G36S+V38M mutations in gp41 (HXB_2_-SIM) featured similar IC_50_ and infectivity levels (data not shown).

### Determination of viral infectivity

10^4^ U87.CD4.CXCR4 or U87.CD4.CCR5 cells seeded in 96-well plates were infected (in triplicate wells) with serial 2-fold dilutions of viral supernatants, (400 to 50 pg/well of p24 antigen). Infection was synchronized by spinoculation (1200×g, 2 hours, 25°C) and monitored after 48 hours by measuring bioluminescence in cell lysates (Promega luciferase assay system) using a Tecan Genios Pro Luminometer. Relative Light Units (RLUs) were plotted against viral p24 inoculi and the slope of the linear regression was calculated using GraphPad Prism v5.01 (GraphPad Software). Relative infectivity (RI) was defined as the ratio of the infectivity (slope) of each recombinant virus to that of the reference strain HXB_2_.

### Enfuvirtide phenotypic susceptibility/resistance testing

10^4^ U87.CD4.CXCR4 or U87.CD4.CCR5 cells were infected with recombinant viral supernatants in the presence of increasing enfuvirtide (Eurogentec) concentrations (0.04 ng/ml to 15×10^3^ ng/ml). Viral input was adjusted to produce 10^5^ RLUs. Infection (in triplicate wells) was synchronized by spinoculation and luciferase activity was monitored 48 hours after infection. IC_50_ values were calculated using the GraphPad Prism software and resistance was expressed as the fold-change in IC_50_ (FCIC_50_) relative to HXB_2_.

### Statistical analyses

RI and FCIC_50_ are expressed as median with the 25^th^ and 75^th^ percentiles [interquartile range, IQR] and p-values<0.05 (two-sided) were considered statistically significant. Intra-patient results were compared using a nonparametric Kruskal-Wallis test followed by Dunn's Multiple Comparison Test. Nonparametrical Mann-Whitney test (U-test) was used to compare inter-patient samples and Wilcoxon signed-rank test to analyze paired Env and HR1–HR2 recombinant viruses. For dual-tropic Env recombinant viruses, mean FCIC_50_s were used.

## Supporting Information

Figure S1 **Construction of the pNL4-3Δ*env*.Luc and the pNL4-3Δ*HR1–HR2*.Luc vectors.** After subloning of the *EcoR*I-*Not*I fragment from pNL4-3.GIV.eGFP into pZero-2, fragments 6220–8322 or 7854–8214 were deleted by inverse PCR and an *Afe*I and *Asc*I restriction site was introduced in each of the constructs respectively. The *Sal*I-*Bam*HI fragments deleted of the Env ectodomain or of the HR1–HR2 regions were cloned into the *Sal*I-*Bam*HI digested pNL4-3.Luc in order to generate the final backbones pNL4-3Δ*env*.Luc and pNL4-3Δ*HR1–HR2*.Luc.
(PDF)

Table S1 **Supporting table**
(PDF)

Table S2 **Supporting table**
(PDF)

## References

[1] Doms RW, Moore JP (2000). HIV-1 membrane fusion: targets of opportunity.. J Cell Biol.

[2] Doms RW, Trono D (2000). The plasma membrane as a combat zone in the HIV battlefield.. Genes Dev.

[3] Eckert DM, Kim PS (2001). Mechanisms of viral membrane fusion and its inhibition.. Annu Rev Biochem.

[4] Sullivan N, Thali M, Furman C, Ho DD, Sodroski J (1993). Effect of amino acid changes in the V1/V2 region of the human immunodeficiency virus type 1 gp120 glycoprotein on subunit association, syncytium formation, and recognition by a neutralizing antibody.. J Virol.

[5] Koito A, Harrowe G, Levy JA, Cheng-Mayer C (1994). Functional role of the V1/V2 region of human immunodeficiency virus type 1 envelope glycoprotein gp120 in infection of primary macrophages and soluble CD4 neutralization.. J Virol.

[6] Groenink M, Fouchier RA, Broersen S, Baker CH, Koot M (1993). Relation of phenotype evolution of HIV-1 to envelope V2 configuration.. Science.

[7] Chan DC, Chutkowski CT, Kim PS (1998). Evidence that a prominent cavity in the coiled coil of HIV type 1 gp41 is an attractive drug target.. Proc Natl Acad Sci U S A.

[8] Markosyan RM, Cohen FS, Melikyan GB (2003). HIV-1 envelope proteins complete their folding into six-helix bundles immediately after fusion pore formation.. Mol Biol Cell.

[9] Melikyan GB, Markosyan RM, Hemmati H, Delmedico MK, Lambert DM (2000). Evidence that the transition of HIV-1 gp41 into a six-helix bundle, not the bundle configuration, induces membrane fusion.. J Cell Biol.

[10] Hanna GJ (2002). HIV-1 genotypic and phenotypic resistance.. Clin Lab Med.

[11] Kilby JM, Hopkins S, Venetta TM, DiMassimo B, Cloud GA (1998). Potent suppression of HIV-1 replication in humans by T-20, a peptide inhibitor of gp41-mediated virus entry.. Nat Med.

[12] Chen SS (1994). Functional role of the zipper motif region of human immunodeficiency virus type 1 transmembrane protein gp41.. J Virol.

[13] Wild CT, Shugars DC, Greenwell TK, McDanal CB, Matthews TJ (1994). Peptides corresponding to a predictive alpha-helical domain of human immunodeficiency virus type 1 gp41 are potent inhibitors of virus infection.. Proc Natl Acad Sci U S A.

[14] Moyle G (2003). Stopping HIV fusion with enfuvirtide: the first step to extracellular HAART.. J Antimicrob Chemother.

[15] Lu J, Deeks SG, Hoh R, Beatty G, Kuritzkes BA (2006). Rapid emergence of enfuvirtide resistance in HIV-1-infected patients: results of a clonal analysis.. J Acquir Immune Defic Syndr.

[16] Labrosse B, Labernardiere JL, Dam E, Trouplin V, Skrabal K (2003). Baseline susceptibility of primary human immunodeficiency virus type 1 to entry inhibitors.. J Virol.

[17] Rimsky LT, Shugars DC, Matthews TJ (1998). Determinants of human immunodeficiency virus type 1 resistance to gp41-derived inhibitory peptides.. J Virol.

[18] Wei X, Decker JM, Liu H, Zhang Z, Arani RB (2002). Emergence of resistant human immunodeficiency virus type 1 in patients receiving fusion inhibitor (T-20) monotherapy.. Antimicrob Agents Chemother.

[19] Xu L, Pozniak A, Wildfire A, Stanfield-Oakley SA, Mosier SM (2005). Emergence and evolution of enfuvirtide resistance following long-term therapy involves heptad repeat 2 mutations within gp41.. Antimicrob Agents Chemother.

[20] Baldwin CE, Berkhout B (2006). Second site escape of a T20-dependent HIV-1 variant by a single amino acid change in the CD4 binding region of the envelope glycoprotein.. Retrovirology.

[21] Chen SS, Lee SF, Hao HJ, Chuang CK (1998). Mutations in the leucine zipper-like heptad repeat sequence of human immunodeficiency virus type 1 gp41 dominantly interfere with wild-type virus infectivity.. J Virol.

[22] Greenberg ML, Cammack N (2004). Resistance to enfuvirtide, the first HIV fusion inhibitor.. J Antimicrob Chemother.

[23] Menzo S, Castagna A, Monachetti A, Hasson H, Danise A (2004). Genotype and phenotype patterns of human immunodeficiency virus type 1 resistance to enfuvirtide during long-term treatment.. Antimicrob Agents Chemother.

[24] Reeves JD, Lee FH, Miamidian JL, Jabara CB, Juntilla MM (2005). Enfuvirtide resistance mutations: impact on human immunodeficiency virus envelope function, entry inhibitor sensitivity, and virus neutralization.. J Virol.

[25] Sista PR, Melby T, Davison D, Jin L, Mosier S (2004). Characterization of determinants of genotypic and phenotypic resistance to enfuvirtide in baseline and on-treatment HIV-1 isolates.. Aids.

[26] Su C, Melby T, DeMasi R, Ravindran P, Heilek-Snyder G (2006). Genotypic changes in human immunodeficiency virus type 1 envelope glycoproteins on treatment with the fusion inhibitor enfuvirtide and their influence on changes in drug susceptibility in vitro.. J Clin Virol.

[27] Melby T, Sista P, DeMasi R, Kirkland T, Roberts N (2006). Characterization of envelope glycoprotein gp41 genotype and phenotypic susceptibility to enfuvirtide at baseline and on treatment in the phase III clinical trials TORO-1 and TORO-2.. AIDS Res Hum Retroviruses.

[28] Lu J, Sista P, Giguel F, Greenberg M, Kuritzkes DR (2004). Relative replicative fitness of human immunodeficiency virus type 1 mutants resistant to enfuvirtide (T-20).. J Virol.

[29] Ray N, Blackburn LA, Doms RW (2009). HR-2 mutations in human immunodeficiency virus type 1 gp41 restore fusion kinetics delayed by HR-1 mutations that cause clinical resistance to enfuvirtide.. J Virol.

[30] Reeves JD, Gallo SA, Ahmad N, Miamidian JL, Harvey PE (2002). Sensitivity of HIV-1 to entry inhibitors correlates with envelope/coreceptor affinity, receptor density, and fusion kinetics.. Proc Natl Acad Sci U S A.

[31] Jenwitheesuk E, Samudrala R (2005). Heptad-repeat-2 mutations enhance the stability of the enfuvirtide-resistant HIV-1 gp41 hairpin structure.. Antivir Ther.

[32] Derdeyn CA, Decker JM, Sfakianos JN, Wu X, O'Brien WA (2000). Sensitivity of human immunodeficiency virus type 1 to the fusion inhibitor T-20 is modulated by coreceptor specificity defined by the V3 loop of gp120.. J Virol.

[33] Derdeyn CA, Decker JM, Sfakianos JN, Zhang Z, O'Brien WA (2001). Sensitivity of human immunodeficiency virus type 1 to fusion inhibitors targeted to the gp41 first heptad repeat involves distinct regions of gp41 and is consistently modulated by gp120 interactions with the coreceptor.. J Virol.

[34] Labrosse B, Morand-Joubert L, Goubard A, Rochas S, Labernardiere JL (2006). Role of the envelope genetic context in the development of enfuvirtide resistance in human immunodeficiency virus type 1-infected patients.. J Virol.

[35] Goubard A, Clavel F, Mammano F, Labrosse B (2009). In vivo selection by enfuvirtide of HIV type-1 env quasispecies with optimal potential for phenotypic expression of HR1 mutations.. Antivir Ther.

[36] Heil ML, Decker JM, Sfakianos JN, Shaw GM, Hunter E (2004). Determinants of human immunodeficiency virus type 1 baseline susceptibility to the fusion inhibitors enfuvirtide and T-649 reside outside the peptide interaction site.. J Virol.

[37] Nameki D, Kodama E, Ikeuchi M, Mabuchi N, Otaka A (2005). Mutations conferring resistance to human immunodeficiency virus type 1 fusion inhibitors are restricted by gp41 and Rev-responsive element functions.. J Virol.

[38] Ray N, Harrison JE, Blackburn LA, Martin JN, Deeks SG (2007). Clinical resistance to enfuvirtide does not affect susceptibility of human immunodeficiency virus type 1 to other classes of entry inhibitors.. J Virol.

[39] Melby T, Demasi R, Cammack N, Miralles GD, Greenberg ML (2007). Evolution of genotypic and phenotypic resistance during chronic treatment with the fusion inhibitor T-1249.. AIDS Res Hum Retroviruses.

[40] Lohrengel S, Hermann F, Hagmann I, Oberwinkler H, Scrivano L (2005). Determinants of human immunodeficiency virus type 1 resistance to membrane-anchored gp41-derived peptides.. J Virol.

[41] Perez-Alvarez L, Carmona R, Ocampo A, Asorey A, Miralles C (2006). Long-term monitoring of genotypic and phenotypic resistance to T20 in treated patients infected with HIV-1.. J Med Virol.

[42] Bai X, Wilson KL, Seedorff JE, Ahrens D, Green J (2008). Impact of the Enfuvirtide Resistance Mutation N43D and the Associated Baseline Polymorphism E137K on Peptide Sensitivity and Six-Helix Bundle Structure.. Biochemistry.

[43] Mink M, Mosier SM, Janumpalli S, Davison D, Jin L (2005). Impact of human immunodeficiency virus type 1 gp41 amino acid substitutions selected during enfuvirtide treatment on gp41 binding and antiviral potency of enfuvirtide in vitro.. J Virol.

[44] Reeves JD, Miamidian JL, Biscone MJ, Lee FH, Ahmad N (2004). Impact of mutations in the coreceptor binding site on human immunodeficiency virus type 1 fusion, infection, and entry inhibitor sensitivity.. J Virol.

[45] Platt EJ, Durnin JP, Kabat D (2005). Kinetic factors control efficiencies of cell entry, efficacies of entry inhibitors, and mechanisms of adaptation of human immunodeficiency virus.. J Virol.

[46] Bjorndal A, Deng H, Jansson M, Fiore JR, Colognesi C (1997). Coreceptor usage of primary human immunodeficiency virus type 1 isolates varies according to biological phenotype.. J Virol.

[47] Boom R, Sol CJ, Salimans MM, Jansen CL, Wertheim-van Dillen PM (1990). Rapid and simple method for purification of nucleic acids.. J Clin Microbiol.

[48] Edgar RC (2004). MUSCLE: multiple sequence alignment with high accuracy and high throughput.. Nucleic Acids Res.

[49] Page KA, Landau NR, Littman DR (1990). Construction and use of a human immunodeficiency virus vector for analysis of virus infectivity.. J Virol.

[50] Gu X, Fu YX, Li WH (1995). Maximum likelihood estimation of the heterogeneity of substitution rate among nucleotide sites.. Mol Biol Evol.

[51] Guindon S, Gascuel O (2003). A simple, fast, and accurate algorithm to estimate large phylogenies by maximum likelihood.. Syst Biol.

[52] Hasegawa M, Kishino H, Yano T (1985). Dating of the human-ape splitting by a molecular clock of mitochondrial DNA.. J Mol Evol.

[53] Roman F, Ammerlaan W, Plesseria JM, Deroo S, Muller CP (2006). A new recombinant virus system for the study of HIV-1 entry and inhibition.. J Virol Methods.

[54] Perez-Bercoff D, Wurtzer S, Compain S, Benech H, Clavel F (2007). Human immunodeficiency virus type 1: resistance to nucleoside analogues and replicative capacity in primary human macrophages.. J Virol.

