# Supplementary figures and images for: Impact of the HIV-1 env Genetic Context outside HR1–HR2 on Resistance to the Fusion Inhibitor Enfuvirtide and Viral Infectivity in Clinical Isolates
Source: PLoS One. 2011 Jul 8;6(7):e21535. doi: 10.1371/journal.pone.0021535 (PMC3132734; doi:10.1371/journal.pone.0021535)

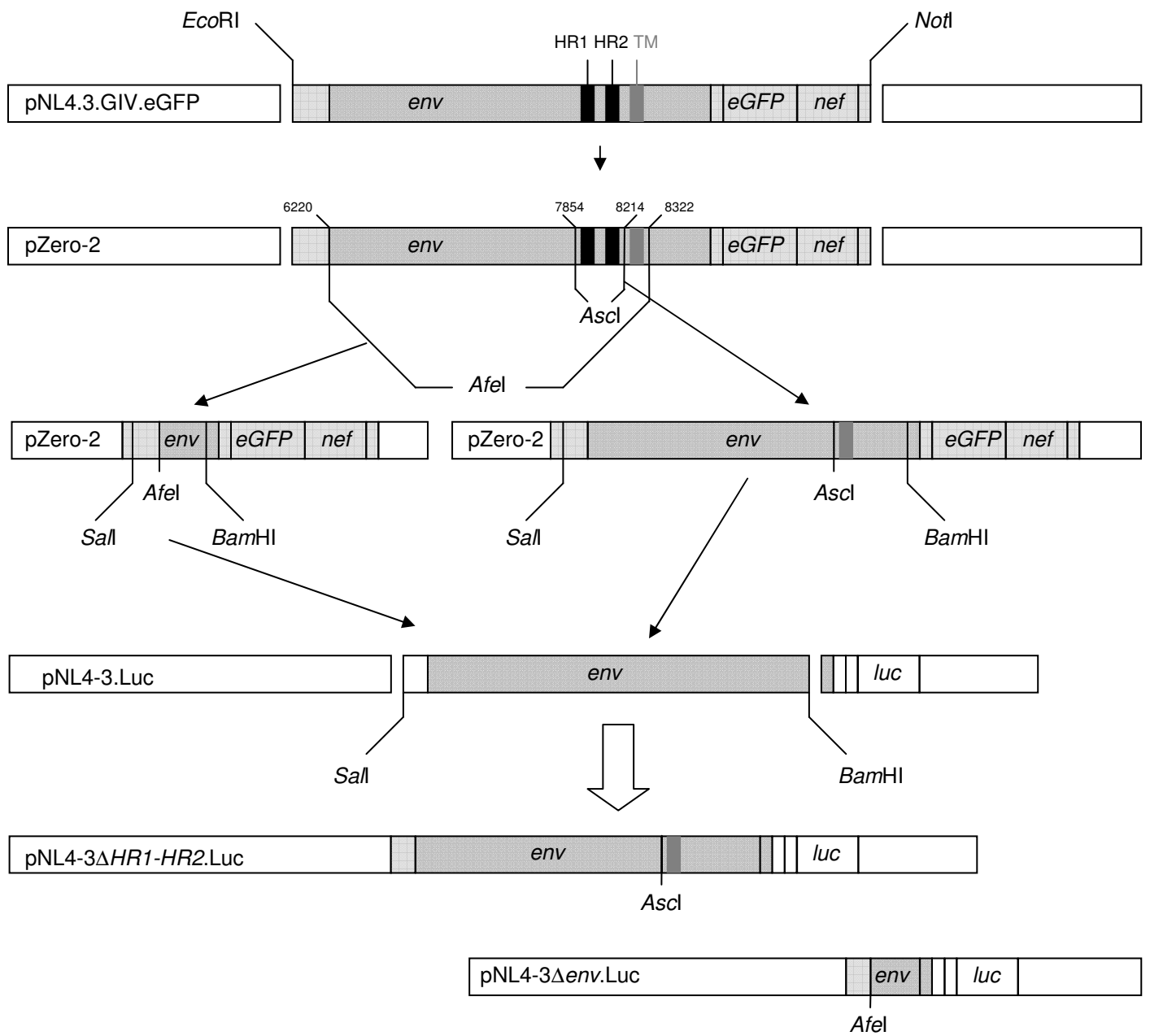

Supplement: Figure S1 — Construction of the pNL4-3Δenv.Luc and the pNL4-3ΔHR1–HR2.Luc vectors. After subloning of the EcoRI-NotI fragment from pNL4-3.GIV.eGFP into pZero-2, fragments 6220–8322 or 7854–8214 were deleted by inverse PCR and an AfeI and AscI restriction site was introduced in each of the constructs respectively. The SalI-BamHI fragments deleted of the Env ectodomain or of the HR1–HR2 regions were cloned into the SalI-BamHI digested pNL4-3.Luc in order to generate the final backbones pNL4-3Δenv.Luc and pNL4-3ΔHR1–HR2.Luc. (PDF) [file pone.0021535.s001.pdf]
