# Supplementary material for: Impact of the HIV-1 env Genetic Context outside HR1–HR2 on Resistance to the Fusion Inhibitor Enfuvirtide and Viral Infectivity in Clinical Isolates
Source: PLoS One. 2011 Jul 8;6(7):e21535. doi: 10.1371/journal.pone.0021535 (PMC3132734; doi:10.1371/journal.pone.0021535)
Supplement: Table S1 — Supporting table (PDF) [file pone.0021535.s002.pdf]

Table S1. Sequence alignments of the HR1 and the HR2 regions

Clone[a]	I[b]/NI[c]	FCIC$_{50}$[d] HR1-HR2	RI[e] HR1-HR2	FCIC$_{50}$ ENV		RI ENV		Amino acid sequences[f]
				CXCR4 cells	CCR5 cells	CXCR4 cells	CCR5 cells	33.....40........50........60........70........80........90.......100........110.......120.......130.......140.......150.
HXB2								LLS**GIVQQQNNLL**RAIEAQQHLLQLTVWGIKQLQARILAVERYLKDQQLLGIWGCSGKLICTTAVPWNASW.SNKSLEQIWNHTTWMEWDREINNYTSLIHSLIEE**S**QNQQEKNEQELLEL
A_-5.2	I	4.14	0.61	17.42	14.0	0.02	0.24	------------------------------------V---------------------H---------T--.--R---Y--SNM---Q-E---D---Q--YT------I------KD----
A_-5.5	I	2.86	0.59	ND[g]	9.19	ND	0.03	------------------------------------V---------------------H---------T--.--R---Y--SNM---Q-----D---Q--YT------I------K-----
A_-5.7	I	3.84	0.61	ND	4.87	ND	0.01	-------------K----------------------V---------------------H---------T--.--R---Y--SNM---Q-----D---Q--YT------I------K-----
A_-5.8	NI	ND	ND	ND	ND	ND	ND	------------------------------------V---------------------H---------T--.--R---Y--SNM---Q-----D---Q--YT------I------K-----
A_-5.14	I	7.16	0.63	ND	12.6	ND	0.10	-----------------------------V------V---------------------H---------T--.--R---Y--SNM---Q-E---D---Q--YT------I------KD----
A_20.1	I	512	1.79	2080	1126	0.03	0.17	V--D---------K----------------------V---------------------H---------T--.--R---Y--ENM---Q-E---D---QV-YT------I------K-----
A_20.3	NI	ND	ND	ND	ND	ND	ND	---E---------K----------------------V---------------------H---------T--.--R--QY--QNM---Q-EK--D------YT------I------K--S--
A_20.7	NI	ND	ND	ND	ND	ND	ND	---V--------------------------------V---------------------H---------T--.--R--AY--QNM---Q-EK--D------YT------I------K-----
A_20.8	NI	ND	ND	ND	ND	ND	ND	V--D---------K----------------------V---------------------H---------T--.--R--AY--QNM---Q-EK--D------YT------I------K-----
A_20.9	NI	ND	ND	ND	ND	ND	ND	V--D---------K----------------------V---------------------H---------T--.--R--AY--QNM---Q-EK--G------YT------I------K-----
A_54.4	I	1062	1.70	2116	1248	0.02	0.26	V--D---------K----------------------V---------------------H---------T--.--R--DY--QNM---Q-E---D------YT------I------K-----
A_54.5	NI	ND	ND	ND	ND	ND	ND	V--D---------K----------------------V---------R-----------H---------T--.--R--AY--QNM-----EK--D---Q--YT------I------K-----
A_54.7	I	741	0.72	ND	924	ND	0.07	V--D---------K----------------------V---------------------H---------T--.--R--DY--QNM---Q-E---D------YT------I------K-----
A_54.8	I	862	0.31	ND	2397	ND	0.14	V--D---------K----------------------V---------------------H---------T--.--R-WAY--QNM---Q-E---D------YT------I------K-----
A_54.9	I	1564	0.58	ND	2002	ND	0.05	V--D---------K----------------------V---------------------H---------T--.--R--AY--QNM-----EK--D---Q--YT------I------K-----
A_95.2	I	1787	0.41	ND	1426	ND	0.22	V--D---------K----------------------V---------------------H---------N--.--R-WAY--QNM---Q-E---D------YT------I------K-----
A_95.8	NI	ND	ND	ND	ND	ND	ND	V--D---------K----------------------V---------------------H---------N--.--R-WAY--QNM---Q-E---D------YT------I------K-----
A_95.9	NI	ND	ND	ND	ND	ND	ND	V--D---------K----------------------V---------------------H---------T--.--RTHDY--QNM---Q-E---D------YT----P-I------K-----
A_95.10	NI	ND	ND	ND	ND	ND	ND	V--D---------K----------------------V---------------------H---------T--.--R-V-Y--ENM---Q-E---D---QV-YT------I------K-----
B_-5.11	I	4.86	1.78	ND	1.40	ND	0.27	---------S-----------M-------V------V----------------------------------GN--TYDE--GNM---Q-EK--D---NT-YN---K------Q--LK----
B_-5.14	I	7.89	1.34	ND	1.85	ND	0.16	---------S-----------M--------------V----------------------------------GN--TYDE--GNM---Q-EK--D---NT-YN---K------Q--LK----
B_-5.15	NI	ND	ND	ND	ND	ND		---------S-----------M--------------V----------------------------------GN--TYDE--GNM---Q-EK--D---NT-YN---K------Q--LK----
B_-5.16	I	6.00	1.03	ND	3.19	ND	0.17	---------S-----------M--------------V----------------------------------GN--TYDE--SNM---Q-EK--D---NT-YN---K------Q--LK----
B_-5.17	I	6.43	1.25	ND	1.30	ND	0.11	---------S-----------M--------------V----------------------------------GN--TYDE--GNM---Q-EK--D---NT-YN-F-K------Q--LK----
B_16.1	I	1212	1.01	ND	172	ND	0.09	---------SD----------M--------------V----------------------------------GN--TYDE--GNM---Q-EK--D---NT-YN---KA-----Q--LK----
B_16.11	NI	ND	ND	ND	ND	ND	ND	---------SD----------M--------------V----------------------------------GN--TYDE--GNM---Q-EK--D---NT-YN---K------Q--LK----
B_16.3	NI	ND	ND	ND	ND	ND	ND	---------SD----------M--P-----------V----------------------------------GN--TYDE--GNM---Q-EK--D---NT-YN---KA-----Q--LK----
B_16.4	I	1581	0.82	ND	122	ND	0.15	---------SD----------M--------------V----------------------------------GN--TYDE--GNM---Q-EK--D---NT-YN---KA-----Q--LK----
B_45.1	NI	ND	ND	ND	ND	ND	ND	---------SD----------M--------------V--L-------------------------------GN--TYDE--GNM---Q-EK--D---NT-YN---K------Q--LK----
B_45.2	NI	ND	ND	ND	ND	ND	ND	----------D----------M--------------V----------------------------------GN--TYDE--DNM---Q-EK--D---NT-YN---KA-----Q--LK----
B.45.3	I	1062	1.68	ND	397	ND	0.54	---------SD----------M--------------V--L-------------------------------GN--TYDE--GNM---Q-EK--D---NT-YN---K------Q--LK----
B_45.4	I	1855	0.67	ND	449	ND	0.20	---------SD----------M--------------V----------------------------------GN--TYDE--GNM---Q-EK--D---NT-YN---KA-----Q--LK----
B_45.5	NI	ND	ND	ND	ND	ND	ND	---------SD----------M--------------V--L-------------------------------GN--TYDE--GNM---Q-EK--D---NT-YN---K------Q--LK----
C_-9.3	I	1.04	0.89	6.86	7.40	1.29	0.24	--A---------------------------------L-------Q-----------------------T--.--RT-DY--GNM---A-E---D----T-YT------I------K-----
C_-9.9	I	2.51	0.44	6.23	2.96	1.21	0.23	--A---------------------------------L-------Q-----------------------T--.--R--NY--DNM---A-E---D----T-YT------I------K-----
C_-9.22	I	0.84	0.57	2.09	2.16	0.87	0.22	--A---------------------------------L-------Q-----------------------T--.--R--NY--GNM---A-E---D----T-YT------I------K-----
C_-9.23	I	1.78	1.17	4.84	3.57	2.93	0.56	--A---------------------------------L-------Q-----------------------T--.--R--NY--ENM---A-E---D----T-YT------I------K-----
C_27.1	I	210	0.77	665	121	1.27	0.28	--A--A------------------------------L-------Q-----------------------T--.--R--NY--ENM---A-E---DH---T-YT------I------K-----
C_27.3	I	30.9	0.64	98.4	79.8	1.12	0.25	--AD--------------------------------L-------Q-----------------------T--.--R--NY--ENM---A-E---D----T-YT------I------K-----
C_27.4	I	68.0	0.64	178	78.1	1.03	0.21	--AD--------------------------------L-------Q-----------------------T--.--R--NY--ENM---A-E---D----T-YT------I------K-----
C_27.5	I	35.8	0.20	147	104	0.78	0.26	--AD--------------------------------L-------Q-----------------------T--.--R--NY--ENM---A-E---D----T-YT------I------K-----
C_27.6	I	17.6	0.54	65.7	58.5	1.19	0.31	--AD--------------------------------L-------Q-----------------------T--.--R--NY--ENM---A-E---D----T-YT------I------K-----
C_59.2	I	599	1.16	1273	1513	1.86	0.46	--A--A-----M------------------------L-------Q-----------------------T--.--R--NY--ENM---A-E---D----I-YT------I------K-----
C_59.3	I	1026	1.08	1811	1058	1.89	0.52	--A--A-----M------------------------L-------Q-----------------------T--.--R--NY--ENM---A-E---D----I-YT------I------K-----
C_59.4	I	547	0.90	1382	915	1.58	0.45	--A--A-----M------------------------L-------Q-----------------------T--.--R--NY--ENM---A-E---D----I-YT------I------K-----
C_59.8	NI	ND	ND	ND	ND	ND	ND	--A--A-----M------------------------L-------Q-----------------------T--.--R--NY--ENM---A-E--ND----I-YT------I------K-----
C_59.9	I	388	0.54	1767	2261	0.90	0.19	--A--A-----M------------------------L-------Q-----------------------T--.--R--NY--ENM---A-E---D----I-YT------IR-----K-----
C_129.1	I	1139	1.21	2188	2660	2.88	0.85	--A--A-----M------------------------L-------Q-----------------------T--.--R--ND--ENM---A-E---D---NI-YT------I------K-----
C_129.3	I	1942	1.32	2544	2300	2.66	0.98	--A--A-----M------------------------L-------Q-----------------------T--.--R--ND--ENM---A-E---D---NI-YT------I------K-----
C_129.5	NI	ND	ND	ND	ND	ND	ND	--A--A-----M------------------------L-------Q-----------------------T--.--R--ND--ENM-R-A-E---D---NI-YT------I------K-----
C_129.6	I	1677	1.40	2524	1713	2.43	0.87	--A--A-----M------------------------L-------Q-----------------------T--.--R--ND--ENM---A-E---D---NI-YT------I------K-----
C_129.7	I	1273	1.30	3344	1719	2.77	1.02	--A--A-----M------------------------L-------Q-----------------------T--.--R--ND--ENM---A-E---D---NI-YT------I------K-----
D_-5.13	I	5.00	0.35	27.5	ND	0.26	ND	-M--Q-------------R----T-------.----IN---DNM---Q--K--S----V-YT------S------------
D_-5.14	NI	ND	ND	ND	ND	ND	ND	------------------------------------V-------Q------------------T-------.----MD---GNM---Q-EK--S------YT------I------------
D_-5.18	I	0.49	0.13	ND	0.91	ND	0.32	------------------------------------V-------Q------------------T-------.----MN---DNM---Q--K--S---G--YT------I-------K----
D_27.3	I	5720	0.20	2411	ND	0.01	ND	-M--------D---------------------------------Q-------------R----T-------.----MN---DNM---Q--K--SK-----YT------S------------

D_27.4	I	4632	0.12	2759	ND	0.13	ND	-M--------D---------------------------------Q-------------R----T-------.----MN---DNM---Q--K---------YT------S------------
D_27.7	I	5419	0.56	2350	ND	0.17	ND	-M--------D---------------------------------Q-------------R----T-------.----MN---DNM---Q--K--SK-----YT------S------------
D_27.9	I	1570	0.18	466	ND	0.11	ND	-M-------DS---------------------------------Q-------------R----T-------.----MN---DNM---Q--K---------YT------S------------
D_27.10	NI	ND	ND	ND	ND	ND	ND	-M-------DS---------------------------------Q-------------R----T-------.----MN---DNM---Q--K---K-----YT------S------------
D_59.1	NI	ND	ND	ND	ND	ND	ND	-M--------D---------------------------------Q-------------R----T-------.----MN---DNMA--Q--K--SK-----YT----A-S------------
D_59.3	I	7750	0.38	2310	ND	0.25	ND	-M--------D---------------------------------Q-------------R----T-------.----MN---DNM---Q--K--SK-----YT----A-S------------
D_59.4	I	2414	0.37	6572	ND	0.18	ND	-M--------D---------------------------------Q-------------R----T-------.----MN---DNM---Q--K--SK-----YT----A-S------------
D_59.5	I	2566	0.44	4446	ND	0.28	ND	-M--------D---------------------------------Q-------------R----T-------.----MN---DNM---Q--K--SK-----YT------SR-----------
D_59.6	I	5682	0.47	2161	ND	0.03	ND	-M--------D---------------------------------Q-------------R----T-------.----MN---DNM---Q--E--SK-----YT----A-S------------
D_94.3	I	7022	0.81	2587	ND	0.42	ND	-M--------D---------------------------------Q-------------R----T-------.----MN---DNM---Q--K--SK-S---YT----A-S------------
D_94.4	NI	ND	ND	ND	ND	ND	ND	-M--------D---------------------P-----------Q-------------R----T-------.----MN---DNM---Q--K--SK-----YT----A-S--D---------
D_94.5	NI	ND	ND	ND	ND	ND	ND	-M--------D---------------------------------Q-------------R----T-------.----MN---DNM---Q--K--SK-----YT----A-S--D---------
D_94.6	NI	ND	ND	ND	ND	ND	ND	-M--------D---------------------------------Q-------------R----T-------.----MN---DNM---Q--K--SK-S---YT----A-S------------
D_94.7	NI	ND	ND	ND	ND	ND	ND	-M--------D---------------------------------Q-------------R----T-------.----MN---DNM---Q--K--SK-S---YT----A-S------------
E_-129.1	I	0.63	1.79	ND	8.52	ND	0.03	---------------------I--------------V--------------------------T-------.-----DE--KNM---Q---------DI-YN-------------------
E_-129.2	NI	ND	ND	ND	ND	ND	ND	---------------------I--------------V--------------------------T-------.-----DE--KNM---Q---------DI-YN-------------------
E_-129.3	NI	ND	ND	ND	ND	ND	ND	---------------------I--------------V--------------------------T-------.-----DE--KNM---Q-G-------DI-YN-------------------
E_-129.4	I	1.02	0.93	ND	5.09	ND	0.03	---------------------I--------------V--------------------------T-------.-----DE--KNM---Q---------DI-YN-------------------
E_-129.5	I	0.47	1.20	ND	3.97	ND	0.05	---------------------I--------------V--------------------------T-------.-----DE--KNM---Q---------DI-YN-------------------
E_42.1	I	683	1.66	ND	1338	ND	0.06	----------D----------I--------------V--------------------------T-------.----QNE---NM---Q---------GI-YN---KA--------------
E_42.2	NI	ND	ND	ND	ND	ND	ND	----------D----------I--------------V--------------------------T-------.----QDE--GNM---Q---------GI-YN---KA--------------
E_42.4	NI	ND	ND	ND	ND	ND	ND	----------D----------I--------------V--------------------------T-------.----QNE---NM---Q---------GI-YN---KA--------------
E_42.6	I	1048	1.86	5836	2924	0.60	0.15	----------D----------I--------------V--------------------------T-------.-----NE--ENM---Q--K------DI-YN---KA--------------
E_42.7	I	1045	0.49	4518	ND	0.31	ND	----------D----------I--------------V--------------------------T-------.----QDE---NM---Q---------GI-YN---KA--------------

[a] Clone identifiers are composed of a letter designating the patient (A-E) followed by a number corresponding to the week of sampling relative to enfuvirtide treatment beginning at week 0 (first number preceding the dot) followed by the clone number

[b] infectious

[c] non infectious

[d] Fold Change in 50% Inhibitory Concentration

[e] Relative Infectivity

[f] HR1 and HR2 domains are underlined and the region between AA 36 to 45, as well as position 138 in HR2 are highlighted in bold letters.

[g] not determined
