# Supplementary material for: Impact of the HIV-1 env Genetic Context outside HR1–HR2 on Resistance to the Fusion Inhibitor Enfuvirtide and Viral Infectivity in Clinical Isolates
Source: PLoS One. 2011 Jul 8;6(7):e21535. doi: 10.1371/journal.pone.0021535 (PMC3132734; doi:10.1371/journal.pone.0021535)
Supplement: Table S2 — Supporting table (PDF) [file pone.0021535.s003.pdf]

Table S2: Patient clinical data

	week	VL	CD4 count	Actual Treatment	previous treatment history	ENF resistance mutations	resistance mutations	predicted active drugs[a]
A	-37				AZT, AZT + 3TC, 3TC + d4T + SQV, d4T + DDI + IDV, AZT + 3TC + NFV + NVP		PR:10I, 33F, 35D, 36L, 43T, 48V, 50V, 54A, 63P, 71I, 77I, 82A, RT: 41L, 67N, 98G, 118I, 184I, 188L, 210W, 215Y, 219S	
	-5	49900	44	DDI + APV + LPV/r				ENF=1 LPV=0 DDI=0.5 TDF=0.5
	20	21700	82	DDI + ENF + LPV/r + TDF		G36D/E/V		
	54	9900	37			G36D		
	95	64000	53			G36D, S138P		
B	-5	372000	30	LPV/r + EFV + TDF	AZT + DDC, AZT + 3TC + SQV, d4T + 3TC + IDV, EFV + d4T + RTV, NFV, NVP, d4T + TZV, APV/r + d4T + 3TC		PR: 10I, 13V, 32I, 46I, 47V, 54M, 63P, 71V, 82A, 90M. RT: 41L, 69S, 74V, 75L, 98G, 103N, 181C, 190A, 210W, 215Y, 68-70 insertion	
	16	>200000	40	LPV/r + ENF + TDF, ENF interrupted wk 22 to week 29		N43D		ENF=1 LPV=0 TDF=0
	25					N43D, S138A	PR: 10I, 13V, 32I, 46I, 47V, 54M, 63P, 71V, 82A, 90M, RT: 41L, 69S, 74V, 75L, 98G, 103N, 181C, 190A, 210W, 215Y, 68-70 insertion	
	45	176000	20			N43D, S138A		
C	-9	>100.000	90		AZT, AZT + DDI, AZT + DDC, DDI + D4T + RTV, D4T + 3TC + IDV, NFV + SQV + D4T, NFV + NVP + D4T			
	-1			ABV + LPV/r + DDI			PR: L10I, K20R, E35D, M36I, K43T, F53L, I54V, H69Y, A71V, V82A, L90M, RT: M41L, D67N, T69D, K70R, L74I, V75T, V90IV, K101Q, Y181C, G190A, T215F, K219Q	3TC=1 TDF=0.5 AZT=1 ENF=1
	27	1000		AZT + 3TC + TDF+ ENF		G36D,V38A		
	59	30000	130			V38A		
	94					V38A	PR: L10I, K20R, 24V, E35D, M36I, K43T, I54V, H69Y, A71V, L90M, RT: M41L, D67N, T69D, K70R, L74I, V90IV, K101Q, M184V, T215F, K219Q	
	129	80000	80			V38A	PR: L10I, K20R, M36I, K43T, I54V, H69Y, A71V, L90M, RT: M41L, D67N, T69D, K70R, V90IV, K101Q, Y181V, M184V, T215F, K219Q	
D	-113				AZT, AZT + DDC, DDI + D4T + IDV		PR: 24I, 35D, 46L, 54V, 71I, 82A, RT: 41L, 67N, 101Q/H, 181C, 184V, 190A, 210W, 215Y, 44D, 219N	ENF=1 TDF=0.5
	-5	100000	160	NFV + 3TC + NVP				
	27	8000	100	TDF + AZT + 3TC + ENF		N42S+N43D+126K, N42D		
	59	10000	10			N43D+N126K+S138A		
	94	20000	80			N43D+S138A		
E	-133	43300	410		AZT + DDC, AZT + DDI, AZT + 3TC + SQV, 3TC + D4T + IDV, DDI + ABV + NVP + SQV/r.			
	-129	4960		TDF + ABV + LPV/r + NVP			PR: 10I, 20R, 35D, 36V, 43T, 46I, 54V, 63P, 71V, 77I, 82T, 84V, RT: 41L, 67N, 70R, 74I, 101E, 179I, 190A, 215F, 219Q	DDI= 0 AZT=0 3TC=1 TDF=0,5 TPVR/r=0 ENF=1
	42	80408	80	DDI + AZT + 3TC + TDF + TPV/r + ENF		N43D, S138A	PR: 10I, 20R, 35D, 43T, 46I, 54V, 63P, 71V, 77I, 82T, 84V, RT: 41L, 67N, 70R, 101E, 179I 184V, 190A, 215F, 219Q, 74I	

[a] The predicted activity for each drug within the enfuvirtide-based regimen was calculated using the Stanford HIVdb-algorithm (www.hivdb.stanford.edu). The level of inferred resistance was scored as follows: 0 for high-level resistance, 0.5 for intermediate resistance or low-level resistance, 1 for potential low-level resistance or susceptible.
